# A novel deletion in *KRT75L4* mediates the frizzle trait in a Chinese indigenous chicken

**DOI:** 10.1186/s12711-018-0441-7

**Published:** 2018-12-20

**Authors:** Jing Dong, Chuan He, Zhibing Wang, Yanqing Li, Shanshan Li, Lin Tao, Jiebo Chen, Donghua Li, Fenxia Yang, Naibin Li, Quan Zhang, Li Zhang, Guangqin Wang, Fisayo Akinyemi, He Meng, Bingwang Du

**Affiliations:** 1 Animal Science Department of Agricultural College, Guangdong Ocean University, Huguangyan East, Zhanjiang, 524088 Guangdong China; 2 Department of Animal Science, School of Agriculture and Biology, Shanghai Jiao Tong University, Shanghai Key Laboratory of Veterinary Biotechnology, Shanghai, 200240 China; 3 Zhanjiang Jinsheng Animal Husbandry Science and Technology Ltd., Zhanjiang, 524025 Guangdong China

## Abstract

**Background:**

Highly diversified in morphology and structure, feathers have evolved into various forms. Frizzle feathers, which result from a developmental defect of the feather, are observed in several domestic chicken breeds. The frizzle phenotype is consistent with incomplete dominance of a major gene, but the molecular mechanisms that underlie this phenotype remain obscure. Kirin, a Chinese indigenous chicken breed that originated in the Guangdong province, is famous for its frizzle feathers. The *KRT75* gene is considered as the dominant gene responsible for the frizzle trait in several chicken breeds, but this is not the case in the Kirin breed. Thus, the objective of our study was to investigate the genomic region and mutation responsible for this phenotype in this particular breed.

**Results:**

A resource population was produced by crossing Kirin and Huaixiang chickens to produce F_1_ and F_2_ generations. DNA samples from 75 frizzle feather and normal feather individuals were sequenced with double-digest genotyping by sequencing (dd-GBS). After the detection of 525,561 high-quality variants, a genome-wide association analysis was carried out and the gene responsible for the frizzle phenotype was localized within the type II α-keratin cluster on chromosome 33. Sanger sequencing was used to screen for mutations in the exons of five genes of this type II α-keratin cluster. A 15-bp deletion in exon 3 of *KRT75L4* that showed complete segregation with the frizzle phenotype was detected within the F_2_ population. Transcriptome sequencing demonstrated that *KRT75L4* was expressed but that the transcript was shorter in Kirin than in Huaixiang chickens. In addition, by using Sanger sequencing, we were able to confirm that the deletion was in complete linkage with frizzle feathers.

**Conclusions:**

A deletion in the *KRT75L4* gene is responsible for the frizzle feather phenotype in the Kirin chicken. The identification of this mutation, which causes a developmental defect of avian integument appendages, will improve our understanding of the mechanisms that are involved in feather formation.

**Electronic supplementary material:**

The online version of this article (10.1186/s12711-018-0441-7) contains supplementary material, which is available to authorized users.

## Background

Birds display a high degree of diversity in feather structure. In chickens, the frizzle trait is a varietal characteristic, and birds that carry this trait have all their contour feathers curling outward and upward [1, 2]. The rachises of frizzle and normal feathers are different i.e., frizzle feathers turn outward relative to the skin, whereas normal body and flight feathers have a dorsal orientation. In frizzle chickens, rectrices and remiges are less affected but have an irregular appearance, and display other modifications, such as thickening of the barbs and barbules, alteration of the hooklets and in some cases, other structural abnormalities [3, 4]. These modifications of the feather structure reduce the insulating effect of feathers [5–7].

To date, the frizzle mutation has been reported to occur in a single autosomal gene, denoted as the F gene (see [5]), and shows incomplete dominance inheritance [4, 5]. Although the curling of feathers of the frizzle phenotype is visible in heterozygous individuals, it is even more pronounced in homozygous individuals, but frizzle feathers are more ornamental (the curve is less severe and feathers look like daisy petals) in heterozygous than in homozygous chickens. Because of their enhanced heat dissipation under tropical conditions, both homozygous and heterozygous frizzle chickens have a relatively higher meat and egg production than wild type individuals [8, 9].

**Fig. 1:**
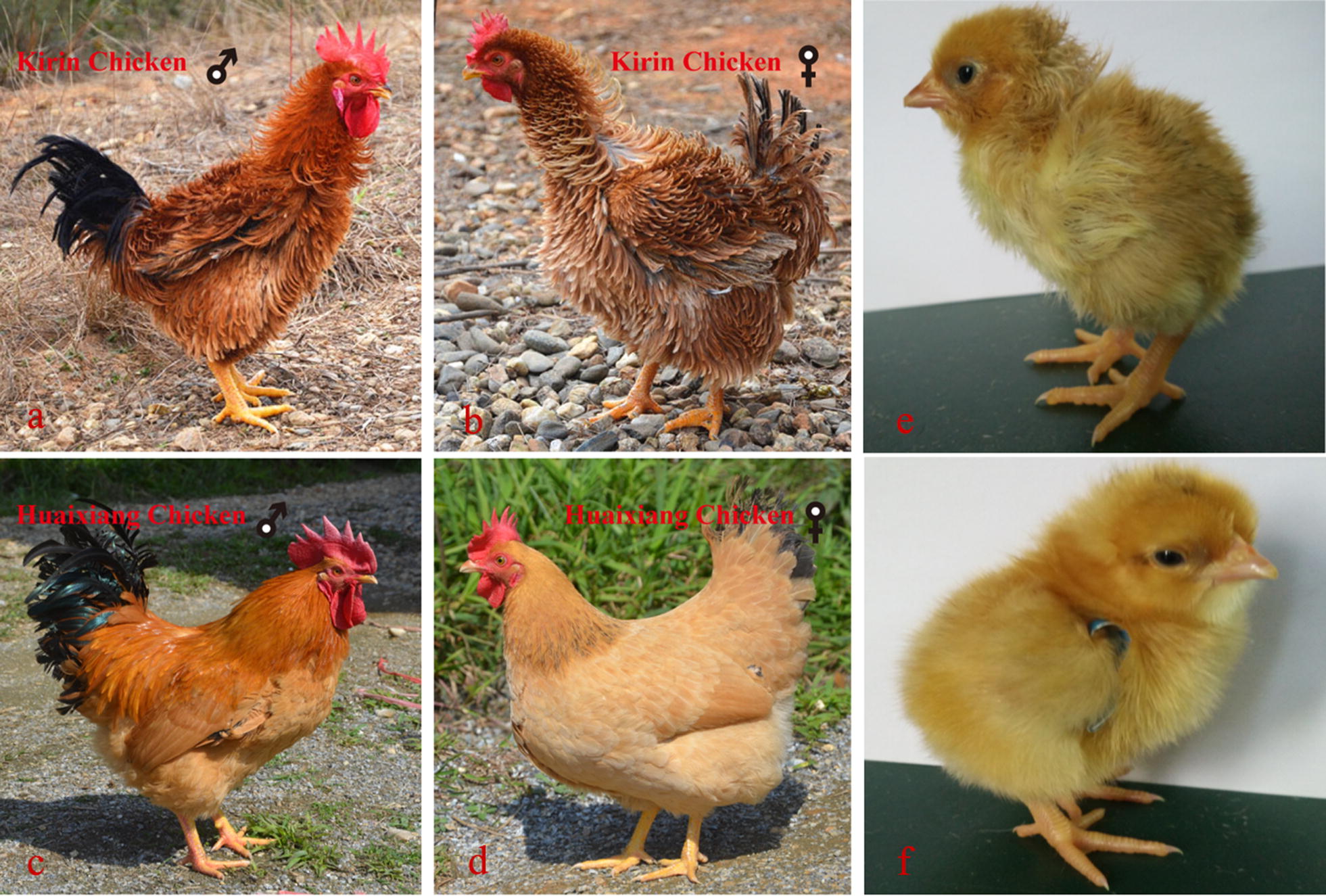
Comparison between frizzle and normal phenotypes. **a**–**d** Adult Kirin (frizzle feathers) and Huaixiang (normal feathers) chickens. Adult frizzle feathers curve away from the body, which gives a highly divergent appearance from normal feathers. **e** One-day-old Kirin and **f** Huaixiang chickens. The downy feathers appear frizzled, which contrasts with feathers from previously reported breeds

Kirin chicken (KRC) is a Chinese indigenous breed of chicken that is noted for its frizzle feathers. The KRC breed originates from the Guangdong province, and is raised exclusively in this region with a warm and humid subtropical climate. Adult KRC have a distinct disorientation of feathers (Fig. 1a–d). In general, the frizzle phenotype is not observed in the down of frizzle dwarf chicken at hatch but becomes more obvious in the second-generation bilateral feathers [10]. In contrast, in KRC, Tao et al. [11] observed the presence of frizzle downy feathers in chicks at hatch (Fig. 1e, f), which suggests a different mechanism for the frizzle trait in this breed. Ng et al. [10] demonstrated that a deletion at the junction between exon 5 and intron 5 of the *KRT75* gene causes the frizzle feather phenotype in frizzle dwarf chicken, but this deletion is absent in KRC, which suggests that another gene is involved in the formation of feathers [10–12] and that the molecular mechanism responsible for frizzle feathers in KRC is different.

To dissect the genetic basis of the frizzle phenotype in KRC, we crossed homozygous frizzle KRC with normal feathered Huaixiang chickens (HX). A double-digest genotyping by sequencing (dd-GBS) based [13] genome-wide association analysis on individuals from the F_2_ generation, allowed us to identify a candidate region for the *F* gene, in KRC. Then, we used Sanger sequencing to ascertain the causative mutation in several candidate genes. We also compared the transcriptome profiles of KRC and HX. Finally, we validated our finding in other breeds, e.g. Houdan (H), Luhua (L) and HX.

## Methods

### Animals and dd-GBS sequencing

A three-generation resource population was produced by crossing purebred KRC with frizzle feathers and HX with normal feathers. The F_1_ generation was derived by mating one KRC male with 10 HX females (KRC × HX), and by mating one HX male with 10 KRC females (HX × KRC). Three male and eight female F_1_ individuals from KRC × HX and three male and eight female F_1_ individuals from HX×KRC were mated, respectively, to produce the F_2_ generation. We conducted a χ^2^-test to study inheritance patterns of the frizzle trait by using the R package software [14].

Eighteen F_1_ and 57 F_2_ homozygous frizzle feather and normal feather individuals were sampled from 12 families [see Additional file 1: Table S1]. Genomic DNA was extracted following the instructions of the AxyPrep™ DNA Gel Extraction kit, double-digested with different restriction enzyme pairs and sequenced. We found that digestion with the enzyme combination, *Pst* I and *Msp* I, was appropriate and highly consistent with in silico digestion-site prediction. Thus, these two restriction enzymes were used to digest total DNA. Adapters marked with specific barcodes were ligated to the restriction-digested overhanging sequences by T4 ligase, following the dd-GBS protocol [13]. DNA samples from the above 75 individuals, marked by different barcode adapters, were mixed and purified according to the manufacturer’s instructions. DNA fragments that carried ligated adapters were amplified with primers that had complementary sequences for each adapter. The constructed 2 × 150 bp libraries were sequenced by the Illumina HiSeq 2000 sequencing system in Shanghai Personal Biotechnology Co., Ltd.

All raw sequences are deposited at NCBI under the BioProject registration number PRJNA445355. Removal of adapter sequences from the reads was done by using AdapterRemoval v2 [15] and the trimmed reads were mapped to the reference genome of *Gallus gallus* (galGal5) by the Burrows–Wheeler Alignment (BWA) tool v0.7.12 [16] in order to identify variants. High-confidence single nucleotide polymorphisms (SNPs) were called by using the GATK software [17], and the outputs were transformed into PED and MAP files format, which were the standard input files of PLINK-1.9 [18].

### Genome-wide association analysis

The dd-GBS based SNPs were filtered out based on the following criteria: (1) minor allele frequency lower than 0.05; (2) SNP missing rate across all individuals higher than 0.2; (3) *P* value for Hardy–Weinberg equilibrium less than 1 × 10e^−40^; and (4) Mendelian errors based on parental genotypes. GWAS on 57 F_2_ samples were performed by using the case-control model in PLINK.

### Sanger sequencing

In order to detect all possible causative mutations in five keratin genes (*KRT5*, *KRT6A*, *KRT75*, *KRT75L2* and *KRT75L4*), we used exon sequencing. Primers for these candidate genes were designed by using primer premier 5.0 and are provided in Additional file 2: Table S2. DNA obtained from blood samples of five homozygous normal and five frizzle feather individuals was pooled, PCR-amplified and sequenced, respectively, with three replicates for each pool.

To validate that the mutation was in exon 3 of the *KRT75L4* gene, blood was sampled from individuals that were randomly selected from KRC, HX, H and L chicken. Heterozygous frizzle samples (F) were obtained from crosses between KRC and other breeds with normal feather breeds. DNA was extracted and pooled from 49 H, 34 HX, 12 L, 86 F and 104 KRC chickens, resulting in five pools. DNA samples of 10 H, 10 HX, 10 L, 40 F and 102 KRC chickens were PCR-amplified individually. PCR conditions were as follows: 5 min at 94 °C for one cycle, and 30 s at 94 °C, 30 s at 60 °C, and 30 s at 72 °C for 35 cycles. The PCR products were sequenced on an ABI 3730 sequencer.

### Transcriptome analysis of frizzle and normal Kirin chicken

We performed the transcriptome analysis of follicle tissues from three KRC and three HX individuals at the embryonic age of E13. Under an RNase-free laminar flow cabinet, feather follicles with skin were removed surgically from the back of each individual, sprayed with RNAsafety, and immediately frozen in liquid nitrogen. Total RNA was extracted with Trizol reagent, and mRNA sequencing was carried out using poly (A)-enriched mRNA. Each of these mRNA samples was converted first into barcoded RNA-seq libraries, and then sequenced on an Illumina HiSeq 2000 sequencer.

The FASTQ sequence of each library was submitted to NCBI (PRJNA445349). Using the Tophat2 [19] program with default parameters, stock-specific transcripts were mapped to the chicken reference genome. The normalized gene expression levels were measured in fragments per kb of exon per million fragments mapped (FPKM) using Cufflinks. Differentially expressed genes (DEG) were identified using the DESeq package [20] based on a *P* value less than 0.05 and |log2(K/H)| higher or equal to  1, where K and H denote the expression level of each gene in the KRC and HX groups, respectively.

## Results

### Resource population from crossbreeding between KRC and HX

Reciprocal matings between homozygous KRC and HX resulted in a heterozygous F_1_, and, as previously reported [3–5], the frizzle phenotype was incompletely penetrant in this population. Penetrance was similar regardless of whether the *F* gene was inherited from a cockerel or a hen, thus ruling out sex-linked inheritance. In order to determine the gene underlying the frizzle trait, six males and 16 females were selected from the F_1_ generation to produce 486 F_2_ offspring. Of these, 113 individuals were homozygous frizzle, 241 were heterozygous frizzle and 132 were homozygous normal feathered. The χ^2^-test (*df* = 2) showed that the frizzle phenotype is consistent with Mendelian inheritance (*P *= 0.22) and incompletely dominant in KRC.

### QTL mapping by genome-wide association analysis

dd-GBS sequencing applied to 75 F_1_ and F_2_ individuals generated 1464 million reads for an average of 3× sequencing depth. After filtering, these reads were aligned to the reference genome, generating about 100,000 effective fragments, which is equivalent to 30 Mb and represents 2.8% of the entire genome with an average depth of 54×. A total of 1,948,658 raw variants were identified, loaded into PLINK 1.9 [18], and filtered according to the criteria presented in the Methods section, which yielded 525,561 variants. The distribution of these variants across the chromosomes was relatively even [see Additional file 3: Figure S1]. Eighteen of the 75 F_1_ individuals were used as references to remove variants with Mendelian errors.

**Fig. 2:**
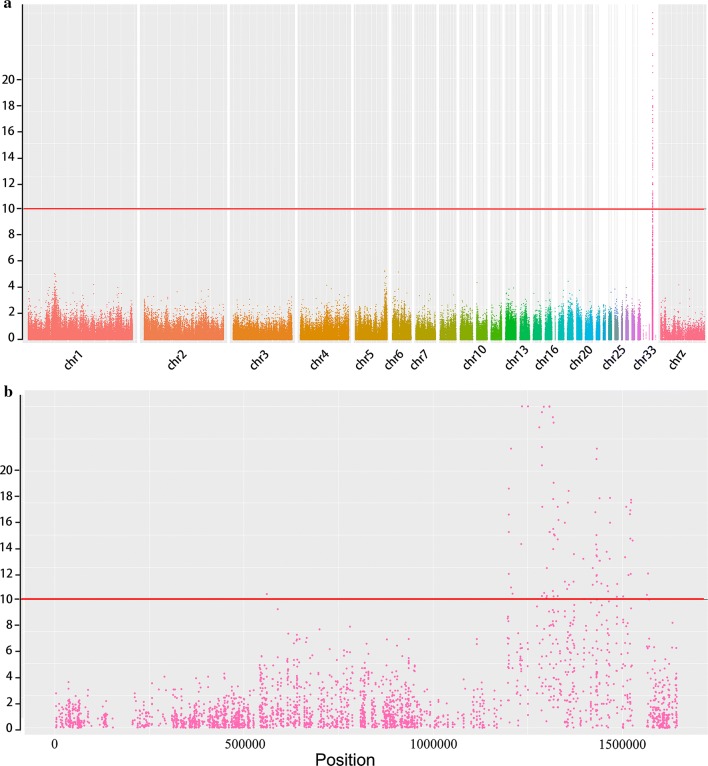
Genome-wide significance of associations. **a** Manhattan plot showing the association of all SNPs with the frizzle feather phenotype. Chromosomes 1–33, W and Z are in different colors. The red solid line indicates a significant association with a *P* value of 1e−10. **b** Manhattan plot of chromosome 33. The red solid line indicates a significant association with a *P* value of 1e−10

We applied a case-control model to calculate the association of variants with the frizzle phenotype across the whole genome (see Fig. 2). SNPs that showed a significant association and their nearest gene are listed in Table 1. A majority of the most significant SNPs were located within a region of 130 kb (*P *= 9.46 × 10^−26^), which corresponds to intron 5 of the *keratin 75*-*like 4* gene (*KRT75L4*). The other most significant SNPs were located in the vicinity of the *KRT75L2*, *KRT5*, *KRT7* and *KRT6A* genes, which encompass a region between 1.19 and 1.52 Mb on chromosome 33 and are putative candidates for the *F* locus. In summary, the candidate QTL overlaps (1.19–1.39 Mb) with the cluster of α-keratin genes on chromosome 33 [21]. To our knowledge, the other detected genes, such as *FAIM2*, *AQP5* and *GALNT6* are not involved in feather formation.

**Table 1:** Markers that are associated with frizzle feather

Chromosome	Position	*P* value	Nearest gene	Distance
33	1206216	1.79E−22	KRT75L1	5183
33	1235337	9.46E−26	KRT75L2	2919
33	1250655	9.46E−26	LOC431300	0
33	1280282	4.09E−24	LOC768978	0
33	1287383	2.60E−25	KRT75L4	5598
33	1287932	3.75E−21	KRT75L4	5049
33	1287940	3.75E−21	KRT75L4	5041
33	1288000	1.38E−22	KRT75L4	4981
33	1288087	6.19E−18	KRT75L4	4894
33	1292993	9.80E−26	KRT75L4	0
33	1307081	9.46E−26	KRT75L4	0
33	1307123	9.46E−26	KRT75L4	0
33	1307957	9.80E−26	KRT75L4	0
33	1316459	6.42E−25	KRT5	0
33	1317239	1.54E−18	KRT5	0
33	1318080	8.31E−20	KRT5	0
33	1318882	1.76E−24	KRT5	0
33	1318889	1.76E−24	KRT5	0
33	1318898	1.76E−24	KRT5	0
33	1328936	6.19E−18	KRT6A	0
33	1356055	2.78E−18	KRT7	0
33	1356078	2.78E−18	KRT7	0
33	1356154	2.78E−18	KRT7	0
33	1357751	3.65E−19	KRT7	0
33	1432037	1.18E−21	FAIM2	0
33	1432581	1.79E−22	FAIM2	0
33	1432677	1.79E−22	FAIM2	0
33	1439835	1.33E−18	FAIM2	4398
33	1467224	1.25E−18	AQP5	3314
33	1510761	6.15E−18	LOC100858973	0
33	1522910	1.75E−18	GALNT6	0
33	1523316	2.78E−18	GALNT6	0

GWAS identified multiple significant markers. For each marker, position information (chromosome and position), significances (*P* value) for each genotype at the position, the nearest gene and distances from corresponding markers are provided

### Identification of a mutation in *KRT75L4* in the KRC

**Fig. 3:**
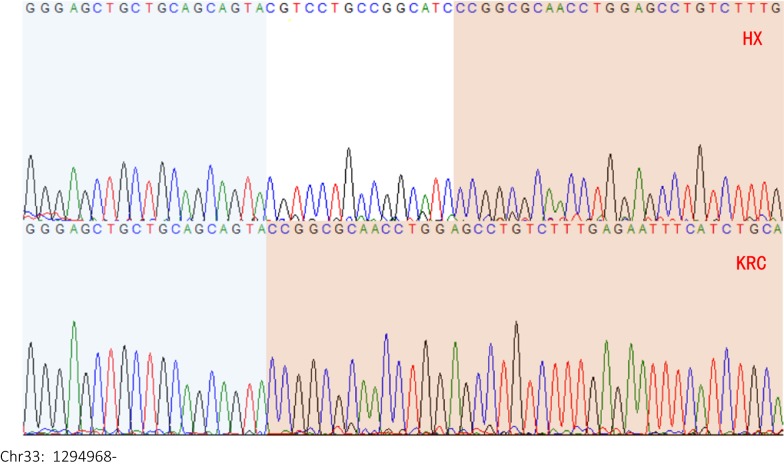
Fluorescent peaks of the 15-bp deletion and its flanking sequences. The sequence on chromosome 33 starts at position 1,294,968 bp of the reference genome (Gallus_gallus-5.0). The upper and lower sequences are from HX and KRC individuals, respectively, with a deletion of 15 bp in KRC. The upstream and downstream sequences of the deletion have blue and red backgrounds, respectively

The candidate region identified by GWAS lies within a genomic interval on chromosome 33, which covers the cluster of type II α-keratin genes. Although in frizzle dwarf chicken, the causative mutation for the frizzle phenotype is localized in the *KRT75* gene [10], previous results indicate that this is not the case in KRC [11]. To detect the putative causative mutations in KRC, we PCR-amplified and sequenced all the exons of five keratin genes (*KRT5*, *KRT6A*, *KRT75*, *KRT75L2*, and *KRT75L4*) located within the region detected by GWAS. The *KRT75L4* gene has three isoforms (ENSGALT00000086819, ENSGALT00000090244 and ENSGALT00000047324) and the transcript is associated with two gene entries (ENSGALG00000044875 and ENSGALG00000043689). We analyzed the DNA extracted from 10 homozygous F_2_ individuals, i.e. five with normal and five with frizzle feathers and detected 90 synonymous substitutions, five missense substitutions, two deletions and three insertions (indels). Among these five indels, only one was located within the coding sequence of *KRT75L4*, i.e. a 15-bp deletion (Fig. 3), which can alter the sequence of the corresponding protein. This deletion showed complete segregation with the frizzle phenotype in the F_2_ samples that were sequenced; and thus, was named *F* deletion. All the other variants were excluded because at least one wild type chicken had the same genotype as that in the chickens with frizzle feathers.

### Whole-transcriptome sequencing

Whole-transcriptome sequencing produced an average of 49 million reads for each sample. After the trimming and filtering steps, 37.4 to 53.6 million high-quality sequence reads from each of the individuals remained and were aligned to the reference genome. On average, 88% of the reads were successfully aligned.

Of the 94 significant differentially expressed genes (DEG) based on a |log_2_(K/H)| higher than  1 and a *P* less than 0.05 [see Additional file 4: Table S4], 50 DEG were up-regulated and 44 were down-regulated in KRC. However, none of these DEG were located in the 1.19–1.52 Mb region detected by GWAS on chromosome 33 or in the type II α-keratin cluster.

**Fig. 4:**
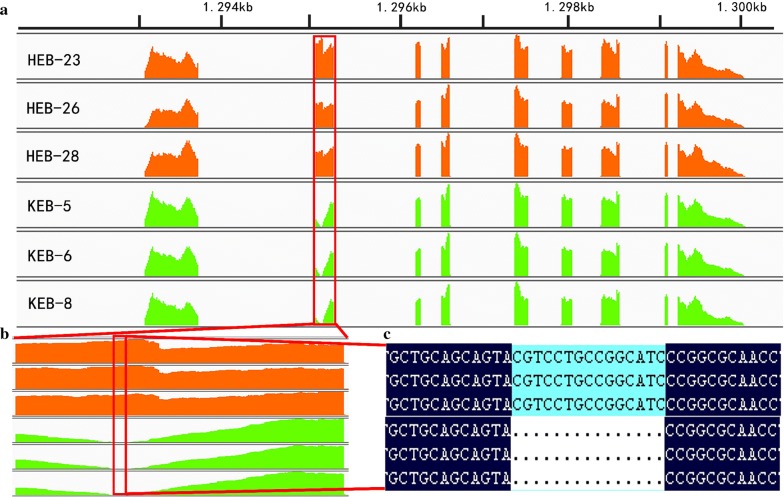
Integrative genomics viewer (IGV) display of KRT75L4 transcripts in KRC and HXC breeds. **a** Part of the alignment tracks of RNA-seq reads in the region of *KRT75L4* is shown. The read coverage tracks of HX and KRC groups are in orange and green, respectively. **b** Zoomed-in image of the second exon in the upper graph. Missing coverage in the middle-front region is observed in the three samples of the KRC group, which is due to the F-deletion that is shown in **c**

The level of expression of *KRT75L4* was similar in the KRC and HX groups. However, the sequence of the *KRT75L4* transcript (displayed by the integrative genomics viewer (IGV) [22, 23]) differed between the KRC and HX groups (Fig. 4). Due to the *F* deletion in one exon (exon 3 of ENSGALT00000090244.1 or exon 4 of ENSGALT00000086819.1), the *KRT75L4* transcript was comparatively shorter in KRC than in HX, leading to the loss of Val-Leu-Pro-Ala-Ser in the translation product, which thus may have a different function compared to the normal protein. The transcripts of the other keratin genes on chromosome 33 were similar for both KRC and HX, which is concordant with the results from the analysis of the KRC’s genome sequence. Thus, our findings indicate that *KRT75L4* is probably the *F* gene.

### Validation in other breeds

To validate furthermore the association detected between *KRT75L4* and the frizzle feather phenotype, we PCR-amplified and sequenced the *F* deletion and its flanking sequences located in *KRT75L4* exon 3 from DNA obtained from other breeds of chickens. The *F* deletion was not found in HX, H and L chickens, which are breeds with normal feathers (Table 2). These results suggest that even if the *F* deletion is not the causative variant, it is strongly linked to the phenotype of frizzle feathers.

**Table 2:** Genotype at the *F*-deletion in the validation groups

	–/–	–/w	w/w
Houdan	0	0	59
Huaixiang	0	0	44
Luhua	0	0	22
Heterozygous F	0	126	0
Kirin	206	0	0

Genoptypes: –/– homozygous *F*-deletion, –/w heterozygous *F*-deletion, w/w homozygous wild type

## Discussion

Heat stress can be a major concern for chickens that are raised in warm climates [24, 25]. A reduction in feather coverage increases heat dissipation and body heat irradiation, and such adaptation to high environmental temperatures has been well-documented [26–28]. Adomako et al. [8] reported favorable effects of the *F* locus on production traits, such as egg weight, egg mass, body weight and productivity index. Thus, identification of the *F* gene is important for breeding programs that are designed to enhance the productive performance of chickens maintained in hot humid climates.

Feathers consist mainly of two types of keratin proteins: α- and β-keratins, which are encoded by multigene families. β-keratins are found only in reptiles and birds, whereas α-keratins exist in all vertebrates [29]. Prum and Williamson [30] attributed the alteration of feather shape to mutations in keratin genes. Cellular and biochemical evidence indicates that α-keratin may have a key role in the early formation of rachides, barbs, and barbules [31]. In addition, the human and mouse homologs of *KRT75*, *KRT6A* and *KRT6B* are expressed in distinct parts of the hair follicle [32–34]. The morphological and structural diversity of feathers probably results from different combinations of α- and β-keratin genes in different intra-feather tissues [21].

Analyses of mouse and human cells showed that some α-keratins are involved in intracellular signaling pathways, and that mutations in the corresponding genes may interfere in the construction of the cell cytoskeleton [35, 36]. Although type II keratin genes are located within a single cluster on human chromosome 12 and mouse chromosome 15 [37], studies on avian keratin genes have been challenging because of their conservation and duplications. In spite of considerable efforts to establish a correspondence between individual α-keratin chicken genes and their homologs in mammals [21], they are not included in the most recent chicken genome assembly and annotation release. Ng et al. [10] reported that the loss of the authentic splice site at the exon5/intron 5 junction of *KRT75* resulted in a 69-bp in-frame deletion within its coding region and played a key role in frizzle feather development. The gene referred to as *KRT75* [10] is actually *KRT6A* according to RefSeq. Furthermore, *KRT75L4* lies within the noncoding region of *KRT6A*, along with *KRT75L1*, *KRT75L2*, *KRT5* and other predicted loci. The official full name of *KRT75L4* is *keratin, type II cytoskeletal 75*-*like 4*, and encodes an uncharacterized protein, with an annotation score of 1 [38]. Our results of transcriptome analysis, combined with other evidence, show that the gene is expressed in chicken tissues, although its function is unknown.

## Conclusions

We have demonstrated that a deletion allele in *KRT75L4* is the major determinant of frizzle feathers in Kirin and, not *KRT6A*, as in other breeds of frizzle chickens. This feather defect seems to be amplified in chickens due to the elaborate morphogenesis of their feathers. Thus, identification of another gene that has a role in the morphology of feathers contributes to a better understanding of the genetics and development of feathers. Finally, our work provides information on a key gene involved in the formation of frizzle feathers and should help to explore furthermore the role of the keratin genes in normal and aberrant vertebrate development.

## Additional files

**Additional file 1: Table S1.** Pedigree and phenotypes of the GWAS population. Description: Columns A and B = sire and dam IDs, respectively; column C = sample IDs; column D = phenotype of the samples.

**Additional file 2: Table S2.** Sequence of the primers used to screen all the exons of the five candidate genes.

**Additional file 3: Figure S1.** Distribution of high-quality SNPs across the whole genome generated by dd-GBS. Each bar represents a chromosome, with a length proportional to the physical length (Mbp) in the Gallus–gallus 5.0 assembly. Yellow and green bars indicate SNP density in 20,000-bp regions.

**Additional file 4: Table S4.** Summary of the differentially expressed genes detected between KRC and HX. Column A = Ensembl IDs of differentially expressed genes; columns B and C = average RPKM of HX and KRC groups, respectively; column D = fold-change both groups; column E = significance P-value.; columns F, G and H = chromosome number and positions of the DEG; column I = gene names.
