# Supplementary figures and images for: A novel deletion in KRT75L4 mediates the frizzle trait in a Chinese indigenous chicken
Source: Genet Sel Evol. 2018 Dec 20;50:68. doi: 10.1186/s12711-018-0441-7 (PMC6302451; doi:10.1186/s12711-018-0441-7)

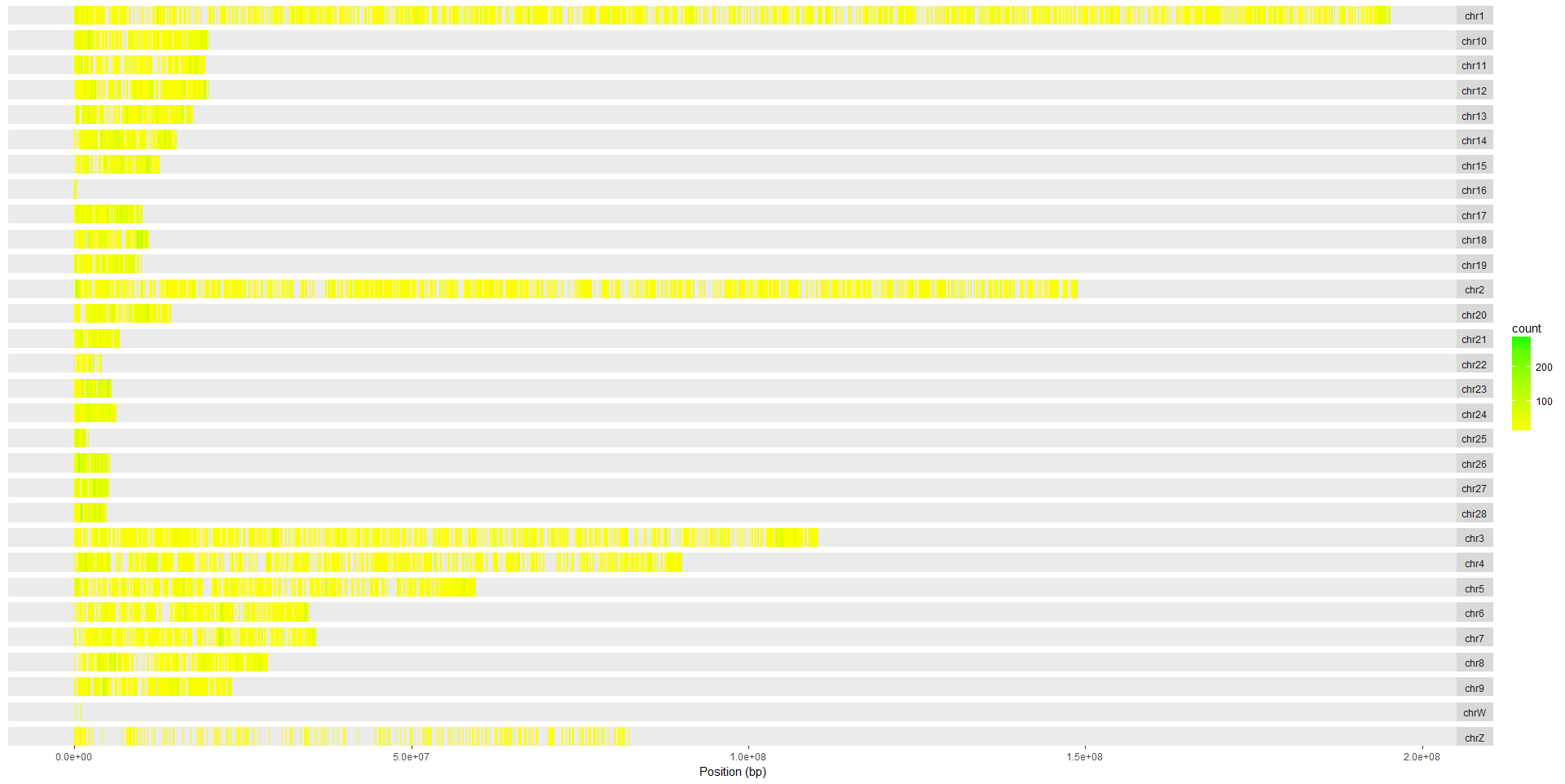

Supplement: Supplementary file 3 — Additional file 3: Figure S1. Distribution of high-quality SNPs across the whole genome generated by dd-GBS. Each bar represents a chromosome, with a length proportional to the physical length (Mbp) in the Gallus–gallus 5.0 assembly. Yellow and green bars indicate SNP density in 20,000-bp regions. [file 12711_2018_441_MOESM3_ESM.png]
